# Sociodemographic factors and research experience impact MD-PhD program acceptance

**DOI:** 10.1172/jci.insight.176146

**Published:** 2024-02-08

**Authors:** Darnell K. Adrian Williams, Briana Christophers, Timothy Keyes, Rachit Kumar, Michael C. Granovetter, Alexandria Adigun, Justin Olivera, Jehron Pura-Bryant, Chynna Smith, Chiemeka Okafor, Mahlet Shibre, Dania Daye, Myles H. Akabas

**Affiliations:** 1 Albert Einstein College of Medicine, Medical Scientist Training Program, Bronx, New York, USA.; 2 Albert Einstein College of Medicine, Bronx, New York, USA.; 3 American Physician Scientists Association, Westford, Massachusetts, USA.; 4 Weill Cornell/Rockefeller/Sloan Kettering Tri-Institutional MD-PhD Program, New York, New York, USA.; 5 Stanford University Medical Scientist Training Program, Stanford, California, USA.; 6 Perelman School of Medicine at the University of Pennsylvania, Medical Scientist Training Program, Philadelphia, Pennsylvania, USA.; 7 University of Pittsburgh–Carnegie Mellon University Medical Scientist Training Program, Pittsburgh, Pennsylvania, USA.; 8 Case Western Reserve University School of Medicine, Cleveland, Ohio, USA.; 9 Harvard Medical School, Boston, Massachusetts, USA.; 10 Division of Interventional Radiology, Massachusetts General Hospital, Boston, Massachusetts, USA.; 11 Departments of Neuroscience and Medicine, Albert Einstein College of Medicine, Bronx, New York, USA.

**Keywords:** Medical statistics

## Abstract

The 2014 NIH Physician-Scientist Workforce Working Group predicted a future shortage of physician-scientists. Subsequent studies have highlighted disparities in MD-PhD admissions based on race, income, and education. Our analysis of data from the Association of American Medical Colleges covering 2014–2021 (15,156 applicants and 6,840 acceptees) revealed that acceptance into US MD-PhD programs correlates with research experience, family income, and research publications. The number of research experiences associated with parental education and family income. Applicants were more likely to be accepted with a family income greater than $50,000 or with one or more publications or presentations. Applicants were less likely to be accepted if they had parents without a graduate degree, were Black/African American, were first-generation college students, or were reapplicants, irrespective of the number of research experiences, publications, or presentations. These findings underscore an admissions bias that favors candidates from affluent and highly educated families, while disadvantaging underrepresented minorities.

## Introduction

Physician-scientists are an integral component of the healthcare and biomedical sciences sector. The combined clinical experience and research training provides a foundation for investigating clinically relevant questions to improve patient outcomes and advance our understanding of the biological basis of disease (1, 2). Over the last 50 years, over 71% of combined MD-PhD program graduates have pursued careers in academia, industry, and government, consistent with their training (3).

Despite the importance of MD-PhD graduates to healthcare and biomedical fields, the 2014 NIH Physician-Scientist Workforce (PSW) Working Group Report found that the number of people working as physician-scientists and entering this career path has decreased (4, 5). In response to the declining number of physician-scientists, the NIH PSW Working Group recommended increasing diversity as a requisite toward building a larger and more productive physician-scientist workforce. Other reports have highlighted how diversity is critical for combating disparities in academia, research, and biomedical careers (6–8). Graduates of MD-PhD programs pursue physician-scientist careers at a rate much higher than their MD- or DO-only counterparts. Therefore, the admissions practices of combined MD-PhD programs must be evaluated through the lens of diversity to assess the impact on the future diversity of the physician-scientist workforce.

Previous studies have reported disparities in MD-PhD program acceptance and matriculation by race/ethnicity, gender, family education, and income (9). In the past six years, 41% of MD-PhD applicants and 49% of matriculants were from families with a household income greater than $100,000; meanwhile, 9% of applicants and 6% of matriculants were from families with a household income of less than $25,000 (10–12). To put this in perspective, the US Federal Reserve reported that in 2022, 30% of adults had a family income less than $25,000 and 32% had an income greater than $100,000. Almost half of MD-PhD matriculants in the last decade had a parent with a doctorate degree (10). Less than one-tenth of MD-PhD matriculants between 2007 and 2012 were first-generation college students (13). From these studies, it is clear that family sociodemographic status is an important determinant of both application to and acceptance into an MD-PhD program.

MD-PhD admissions committees use GPA, Medical College Admissions Test (MCAT) score, research experience, and publications, along with other metrics to screen applications to determine whom to interview and subsequently accept into their programs. Existing literature shows correlations between acceptance, sociodemographic status, and GPA/MCAT scores; however, there is little literature to show that GPA/MCAT scores predict program outcome for MD-PhD trainees. Of note, a previous study showed that medical students with MCAT scores in the middle third of applicant MCAT scores do just as well in medical school as those in the top third of MCAT scores and are a far more diverse group (14). A survey study conducted between 2001 and 2011 indicated that those who engaged in both high school and college research experiences were four times more likely to enter an MD-PhD program rather than an MD-only program (15). This suggests that previous research experience is a significant factor in the decision to apply to an MD-PhD program and in which applicants are invited to interview for MD-PhD programs. If the number of research experiences and publications correlates with family sociodemographic status, then using these metrics as a screening tool for applicants may further homogenize the physician-scientist training pipeline at a time when diversity in thought and experience is more valuable than ever.

With evidence of potential bias in mind, it is crucial for programs to carefully assess whether the metrics they employ for screening and evaluating applicants genuinely reflect indicators of career success or whether they merely mirror and amplify the applicants’ access to socioeconomic opportunities. This self-reflection is essential to ensure a fair and inclusive selection process that does not inadvertently perpetuate disparities and instead fosters a diverse and talented physician-scientist workforce. To aid in this pursuit, it is important to understand the impact of sociodemographic status of applicants on research metrics used to evaluate applicants, just as has been done for the MCAT and GPA.

Here, we investigate the relationships between sociodemographic factors, research involvement, achievements, and likelihood of being accepted into a US MD-PhD program. This approach will highlight contributing factors to the acceptance of applicants to MD-PhD programs and will allow both applicants and programs to target specific disparities that have led to previously identified challenges with diversity in program composition.

## Results

### Demographics

**Figure 1:**
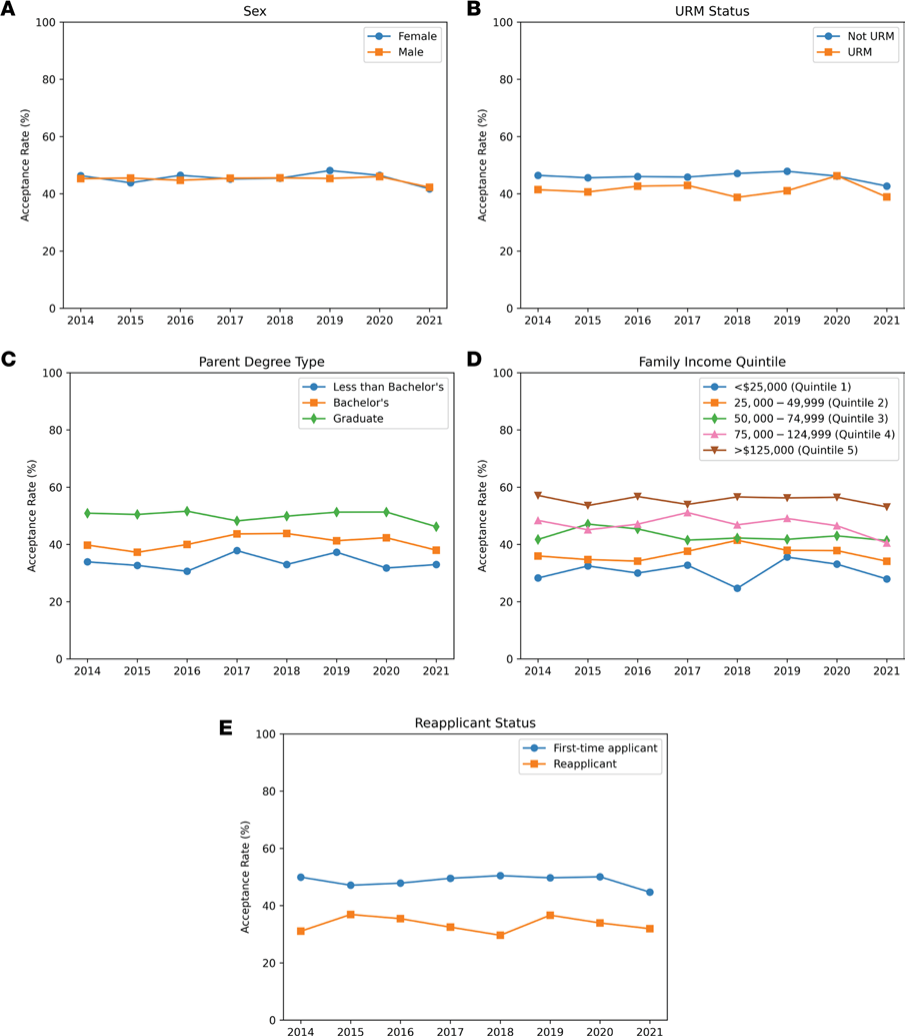
Acceptance rate as a function of demographic group by year of application. (**A**) Acceptance rate by sex. (**B**) Acceptance rate by URM status. (**C**) Acceptance rate in relation to parental degree type. (**D**) Acceptance rate based on family income quintile. (**E**) Acceptance rate based on reapplicant status.

A total of 15,156 applicants and 6,840 acceptees to MD-PhD programs from 2014 to 2021 were included in this study. Individuals who submitted applications in multiple academic years were counted multiple times within the total count of 15,156 applicants. Demographics and acceptance rates are summarized in Table 1. Overall, 45% of applicants (6,822 of 15,156) and 45.2% of acceptees (3,094 of 6,840) were female (Figure 1A); 18% of applicants (2,691 of 15,156) and 16% of acceptees (1,117 of 6,840) were from underrepresented minority (URM) backgrounds (Figure 1B).

First-generation individuals comprised 12.1% (1,832 of 15,156) of applicants and 9.0% (614 of 6,840) of acceptees (Figure 1C). Fifteen percent (2,408 of 15,156) of applicants and 17.9% (1,227 of 6,840) of acceptees had a parent with an MD or MD-PhD degree; 8.6% of applicants (1,309 of 15,156) and 5.8% of acceptees (398 of 6,840) were from the lowest income quintile (Figure 1D). Of all applicants, 22.8% (3,451 of 15,156) had previously applied to MD-PhD programs, but only 16.9% (1,154 of 6,840) of all acceptees had previously applied (Figure 1E).

### Research achievements

**Figure 2:**
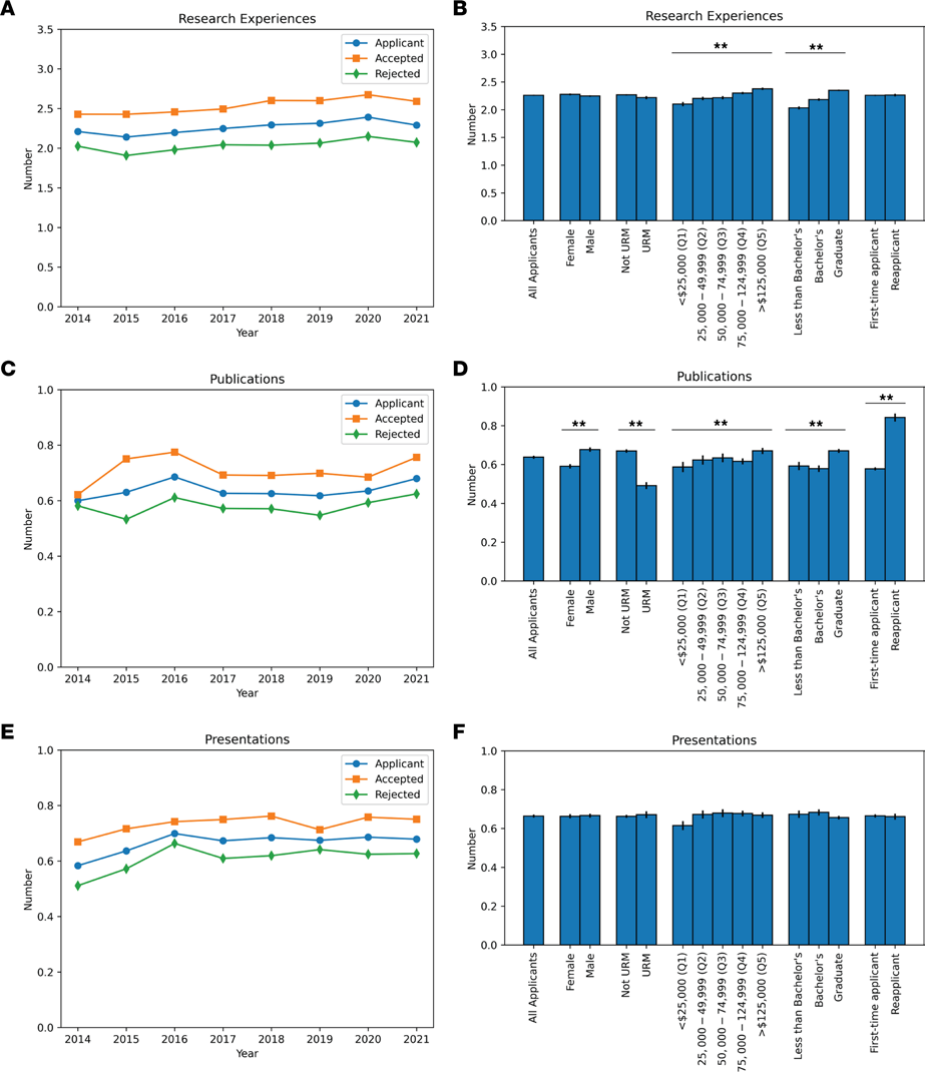
Research achievements of MD-PhD program applicants. (**A**) Mean number of research experiences over the study period. (**B**) Mean number of research experiences by demographic group over the study period. (**C**) Mean number of publications over the study period. (**D**) Mean number of publications by demographic group over the study period. (**E**) Mean number of presentations over the study period. (**F**) Mean number of presentations by demographic group over the study period. **P < 0.01 by ANOVA.

Trends in the mean number of research achievements, including research experience, publications, and presentations, across the study period are shown in Figure 2, A–C. The proportion of applicants with each research achievement is summarized in Table 2.

#### Research experiences.

The mean number of research experiences per applicant was 2.3 (standard deviation [SD] 1.3; Figure 2, A and B).

The number of research experiences did not differ by sex, URM status, or reapplicant status. An ANOVA revealed significant differences in the number of research experiences as a function of the highest education level of the applicants’ parents (*P* < 0.001). Applicants with a graduate school–educated parent had significantly more research experiences (mean 2.4 [SD 1.3]) than those with a college-educated parent (mean 2.2 [SD 1.3], *P* < 0.001) and those who were first-generation (mean 2.0 [SD 1.4], *P* < 0.001); applicants with a college-educated parent also had significantly more research experiences than those who were first-generation (*P* < 0.01). Applicants with higher family income had significantly more research experiences than those in the lowest income quintile by ANOVA (*P* < 0.001; lowest to highest quintile statistics: mean 2.1 [SD 1.4], mean 2.2 [SD 1.4], mean 2.2 [SD 1.3], mean 2.3 [SD 1.3], mean 2.4 [SD 1.3]; all pairwise comparisons significant at *P* < 0.05 except for the comparison of second and third quintile).

#### Publications.

A majority of applicants had no publications (57.8%, 8,755 of 15,156; Table 2 and Figure 2C). The mean number of publications per applicant was 0.6 (SD 1.0, Figure 2D). The proportion of URM applicants (33.7%, 908 of 2,691) with at least one publication was significantly lower compared with non-URM applicants (44.1%, 5,493 of 12,465, *P* < 0.001). URM applicants had a mean of 0.5 publications (SD 0.9), while those who were not URM had a mean of 0.7 publications (SD 1, *P* < 0.001). The mean number of publications for female applicants (0.6 [SD 0.9]) was significantly different from those of male applicants (0.7 [SD 1.0], *P* < 0.001).

A logistic model was fit to predict whether an applicant had at least one publication with parental education level as the predictor (no college degree as baseline); there was no significant effect of having a parent with a college degree on having a publication (*P* = 0.35), although there was a significant effect of having a parent who attended graduate school (*P* < 0.001). However, there was a significant effect of parental education on the total number of publications by ANOVA (*P* < 0.001). Among those who had at least one publication, there was no significant difference in the mean number of publications by parental college education status (*P* = 0.10).

Additionally, a logistic model was fit to predict whether an applicant had at least one publication with income quintile as the predictor (relative to having a parental income within quintile 1). There was only an effect of having a parental income within quintile 5 on having at least one publication (*P* < 0.001). Among those who had at least one publication, an ANOVA revealed no significant difference in the mean number of publications by parental income quintile (*P* = 0.57; lowest to highest quintile statistics: mean 1.5 [SD 0.99], mean 1.5 [SD 1.2], mean 1.5 [SD 1.1], mean 1.5 [SD 1.0], mean 1.5 [SD 0.9]).

Lastly, reapplicants were significantly more likely to have at least one publication (49.9%) than first-time applicants (40.0%, *P* < 0.001). Among those who had at least one publication, applicants who reapplied had a significantly greater number of publications (mean 1.7 [SD 1.3]) than first-time applicants (mean 1.5 [SD 0.9], *P* < 0.001).

#### Presentations.

On average, each applicant had 0.7 (SD 0.9) presentations (Figure 2E). The number of presentations did not vary significantly by any of the demographics examined (Figure 2F).

### Acceptance into MD-PhD programs

**Figure 3:**
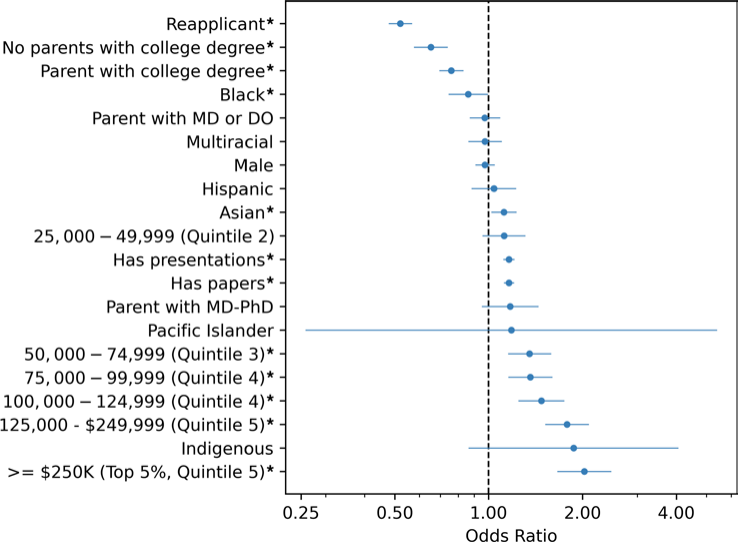
Multivariate model for MD-PhD acceptance. *P < 0.001 by Wald’s test.

The multivariate model reveals income, parental education, race, publications, and presentations impact the odds of acceptance to an MD-PhD program (Table 3 and Figure 3). Those with a childhood family income of greater than $50,000 per year (quintiles 3–5) had higher odds of acceptance, increasing by quintile. Applicants were twice as likely to be accepted if their family income was in the top 5% (greater than or equal to $250,000) compared with those with family income in the lowest quintile (adjusted odds ratio [aOR] 2.025, 95% CI 1.665–2.463, *P* < 0.001). The aOR was 1.12 (95% CI 1.024–1.222, *P* = 0.013) and 0.86 (95% CI 0.747–0.991, *P* = 0.037) for Asian and Black applicants, respectively, compared with White applicants. Having a parent without a graduate degree resulted in lower odds of being accepted, with an aOR of 0.76 (95% CI 0.697–0.827, *P* < 0.001) for those with a parent with a college degree (but no graduate degree) and 0.66 (95% CI 0.578–0.737, *P* < 0.001) for those with parents without a college degree. The strongest negative effect was for reapplicants (aOR 0.521, 95% CI 0.48–0.566, *P* < 0.001). Having at least one publication (aOR 1.16, 95% CI 1.121–1.2, *P* < 0.001) or presentation (aOR 1.16, 95% CI 1.117–1.204, *P* < 0.001) significantly increased the odds of acceptance.

## Discussion

This study highlights recent disparities in the acceptance rates and research achievements of MD-PhD applicants from different demographic groups. First, a significantly smaller percentage of Black/African American applicants gained admission to MD-PhD programs compared with those from other demographics who applied. The acceptance rate has not changed for any sociodemographic group over the eight-year study period despite extensive discussions of the need to diversify the physician-scientist workforce. Second, while the number of research experiences did not differ by URM status, applicants who were first-generation or were at lower family income quintiles had fewer research experiences relative to applicants whose parents attended college or graduate school or were at higher family income quintiles, respectively. Third, applicants who were URM or who had parents without a graduate degree had fewer publications (though not presentations) on average than applicants who were not URM or whose parents did attend graduate school, respectively. Finally, multivariate modeling revealed that applicants at lower income quintiles, Black applicants, first-generation applicants, and reapplicants had lower odds of acceptance to MD-PhD programs; publications and presentations improved odds of acceptance, as did higher family income.

There were no significant differences in sex or overall URM status representation among acceptees compared to the proportion of applicants, consistent with previous work (12). However, the acceptance rate for Black individuals was lower than the overall acceptance rate for all URM individuals and was the only statistically significant difference within that group. We must not discount the barriers that are present based on sex and race/ethnicity in considering research careers, retention in research, and rank of institution attended (16–18). As the disparity in the composition of the current physician-scientist workforce cannot be fully explained by disparities in acceptance of applicants based on these findings, we must look for other explanations so that we can acquire insights from our data. It is likely that those explanations include individuals self-selecting themselves out of applying due to perceived requirements, such as a certain amount of experience, publications, or more resources, for being a competitive applicant.

Research experiences, publications, and presentations were contributors to higher likelihood of acceptance. This finding is consistent with the previously described perception that research experience and publications are needed to gain admission, which dissuades some individuals from applying to MD-PhD programs even if they plan to pursue medical research careers (19). Applicants also cited the belief that multiple research experiences and publications are expected as a reason for pursuing gap years (20). While a survey of MD-PhD directors indicated they do not place a preference on an applicant having publications, more insight is needed into why the number of research achievements, specifically publications, is predictive of acceptance odds (20). In particular, there is notable disparity in the number of research experiences and publications by sociodemographic groups. Another question is whether more extensive research experiences and publications prior to admission increases the likelihood of success in the programs and beyond. However, answers to this question are beyond the scope of the present work. A weakness of the current work is that the duration and depth of research experiences could not be considered, as American Medical College Application Service (AMCAS) application data do not distinguish these factors.

Achievements were not equally distributed by applicant identity, which also provides insight into the access that these individuals have to opportunities. The results of this study clearly show that first-generation and lower-income applicants had fewer research experiences, likely due to lack of social, cultural, and financial capital (10). Previous work has shown that, at the time of taking the MCAT, first-generation students were just as likely to be considering an MD-PhD path as students with college- and graduate-educated parents, yet first-generation applications considering MD-PhD programs were almost one-third less likely to matriculate into an MD-PhD program compared with non–first-generation individuals (13). Minority applicants to medical school are also much less likely to have a family member with an MD, PhD, or both (21). URM individuals are less aware of research careers until later educational stages, and by then, many may consider it too late to redirect their path (2, 21). Early exposure to STEM careers is a key component of gaining admittance to MD-PhD programs (15, 22). Additionally, lower-income individuals may have had less time to engage in research activities if they needed to spend time working during undergraduate training or after graduation (23–25). These research achievements are a key factor in gaining admittance by being a key component of whether people ever apply in the first place. A consistent, data-driven approach is imperative to reduce the economic inequities present in the application process and even before application.

The multivariate model emphasized that Black/African American individuals are less likely to be accepted to MD-PhD programs when all other variables remain equal. This finding persists even when controlling for family income, despite the fact that Black and Hispanic medical students are three times more likely to be from the bottom two income quintiles than White students (26). This finding has persisted despite recent efforts, such as race-blind review and other interventions, to reduce bias. Research has shown that structural racism in the United States has a substantial influence on successful medical school application and on the accumulation of achievements during training; high socioeconomic status is not protective for students of color in the face of structural and interpersonal racism (27). Interestingly, at the time of taking the MCAT, Black individuals are more likely to be considering an MD-PhD program than their White counterparts and are more likely to eventually matriculate into an MD-PhD program if they had considered it at the time of the MCAT (13, 28).

Those who are not accepted on their first attempt often identify areas for improvement in their application prior to reapplying. Reapplicants to medical school who participated in a self-assessment and plan program were more likely to be accepted into medical school their second time applying (29). Transparent and open feedback from advisors and mentors about an applicant’s weaknesses is beneficial in subsequent application cycles; however, having a smaller network may put first-generation individuals at a disadvantage. Community-led organizations and medical student cultural affinity groups, such as the American Physician Scientists Association, Student National Medical Association, and Latino Medical Student Association, also provide a community of like-minded individuals and mentors that, outside of individual MD-PhD programs, can offer support.

The stagnation in MD-PhD program applicants over the past decade necessitates novel outreach strategies (10). A key recommendation is the development of preparatory programs that offer prospective students a glimpse into the life of a physician-scientist by more MD-PhD programs. Preparatory programs should provide mentorship and research opportunities, which are vital for acceptance into MD-PhD programs. Evidence suggests that participation in high school or college research programs increases the likelihood of students pursuing an MD-PhD (22). Successful examples, like the Gateways to the Laboratory at Weill Cornell/Rockefeller/Sloan Kettering Tri-Institutional MD-PhD Program, demonstrate the effectiveness of such initiatives in generating quality applicants (30). Additionally, connecting with summer research programs, especially those targeting URMs, can further this goal. Financial support for low-income undergraduates through work-study or scholarships would encourage long-term mentorship and research publication, key for application success. Regional outreach initiatives, such as the Southeastern Medical Scientist Symposium, have also proven successful in attracting URM students to MD-PhD programs (31). Diversifying the applicant pool further involves reaching out to undergraduate institutions with diverse student bodies, such as historically Black colleges and universities (HBCUs), Hispanic-serving institutions (HSIs), and tribal colleges. Furthermore, collaboration with the NIH to compile resources from various pipeline programs can help identify successful strategies for diversifying the applicant pool.

Moreover, MD-PhD program directors are advised to incorporate holistic review and broaden their focus beyond traditional metrics, such as test scores and undergraduate institution prestige, recognizing the diverse backgrounds and challenges faced by applicants from less prestigious colleges (32). The ambiguous relationship between MCAT scores and future success as a physician-scientist calls for a reduced reliance on rankings and a more nuanced understanding of applicant potential. Holistic review processes in admissions should be critically examined and tailored to each program’s mission and goals, including promoting diversity. This involves considering sociodemographic factors and redefining what constitutes significant research experience (19, 20). Addressing biases in admissions, monitoring applicant demographics for disparities, and offering feedback to applicants, especially from diverse backgrounds, are crucial steps for MD-PhD programs to ensure equity and inclusiveness in their selection process.

### Limitations.

This study has several limitations. We did not examine data from DO-PhD programs, MD-PhD students who decided to pursue a PhD separately from medical school, or MD-PhD programs in other countries. The acceptance rates used in our study account for applicants accepted by an MD-PhD program without accounting for secondary applications, interview invitations, acceptances per applicant, and whether they were accepted to an MD-PhD program but chose not to matriculate or chose to matriculate to an MD program. The deidentified nature of the data prevented tracking individuals, so changes in research achievements between cycles are unknown. The model does not control for an applicant’s GPA, MCAT score, age, geographic location, extracurricular activities, quality of essays, letters of recommendation, or undergraduate institution; these factors contribute to holistic admissions strategies that have had success in diversifying medical school classes (32). This study does not encompass all physician-scientists, as many MDs and DOs go on to become successful physician-scientists without enrolling in PhD programs. Importantly, we are unable to explore the characteristics of people who decided to forgo applying to MD-PhD programs and instead applied to MD-only programs or not at all.

### Conclusions.

With greater outreach to more diverse undergraduate institutions and a continued emphasis on holistic admissions processes, MD-PhD programs can begin to better account for existing systemic imbalances that affect minoritized groups. Recruitment of individuals from historically marginalized backgrounds will require concerted efforts in addition to taking their background into account during the admissions process. These efforts will need to be in concert with creating more opportunities for early exposure to the physician-scientist career path for those from minoritized backgrounds. Viewing research achievements as metrics of success should be reevaluated in the context of potential advantages linked to income and parental education, and program directors should be aware of how using these metrics to screen applicants can further perpetuate the disparities reported in this study. Successful completion of an MD-PhD program is linked to resilience, finances, access to mentors, and other factors from an individual’s unique lived experiences. Program directors and admissions committees should be especially cognizant when considering applicants from lower-income households, those who are first-generation students, or those who are affected by structural racism and bias both before and during the application process itself. We believe doing so will lead to measurable strides in achieving the two-fold goal articulated by the PSW of diversifying and ensuring a sustainable physician-scientist workforce.

## Methods

### Data and participants.

Data from the Association of American Medical Colleges (AAMC) were used for this retrospective cross-sectional study on US MD-PhD program applicants and acceptees from application years 2014 to 2021. The following information was requested per applicant: sex; race/ethnicity; family income; parental education; MD-PhD program acceptance, reapplicant status; and numbers of research experiences, publications, and presentations. Note that for this analysis, program acceptance, not matriculation, was used to focus on the decisions made by the programs rather than by the applicants. Analysis was conducted according to Strengthening the Reporting of Observational Studies in Epidemiology (STROBE) guidelines, and the study was found to be exempt by the Mass General Brigham Institutional Review Board (33).

### Student sociodemographic characteristics.

Applicants who are US citizens and permanent residents self-report race and ethnicity using standardized codes: Asian, American Indian/Alaska Native, Black/African American, Hispanic/Latino, Native Hawaiian/Pacific Islander, White, and Other. Racial and ethnic categories included all individuals who indicated a particular race or ethnicity alone or in combination. URM refers to those from a racial or ethnic group underrepresented in medicine: American Indian/Alaska Native, Black/African American, Hispanic/Latino, and Native Hawaiian/Pacific Islander (34). Codes were not mutually exclusive, so those who reported two or more were categorized as “Multiracial” in multivariate analyses.

We coded parental educational attainment as the maximum degree of education either parent received. In addition, we created two binary variables based on each applicant’s parental educational attainment, indicating whether either of an applicant’s parents obtained a college degree or went to graduate school. We use the term first-generation to refer to applicants with no parents with a college degree (Associate’s or Bachelor’s degrees). For this study, “reapplicant” refers to an individual who previously applied to any US MD-granting medical school program and, therefore, was not limited to only MD-PhD applications.

For our analyses, the family income categories were collapsed into the following: less than $25,000 (quintile 1), $25,000–$49,999 (quintile 2), $50,000–$74,999 (quintile 3), $75,000–$124,999 (quintile 4), and greater than $125,000 (quintile 5). Families with incomes above $250,000 were in the top 5%. Income quintiles were assigned to each applicant by matching the midpoint of the income range reported in their application to the income quintile ranges established in a previously published AAMC study (35).

### Research achievements.

In the data provided by the AAMC from applicants’ AMCAS applications, research experiences are self-reported and categorized by applicants. Applicants may report summer research experiences, meaningful time spent in a research laboratory group, and time spent working on a research project as experiences in their application. Publications include published peer-reviewed research articles, reviews, and book chapters. Presentations refer to all oral and/or poster research presentations.

### Statistics and multivariate modeling.

Univariate summaries of the study cohort were computed and compared across the applicant and acceptee pools. χ^2^ analyses were used for categorical variables, and 2-sample Student’s *t* tests were used for continuous variables with a multiple-hypothesis correction using the Benjamini-Hochberg procedure for controlling the false-discovery rate (36). For all analyses, each application was considered unique, even if it was submitted by a reapplicant. A multivariate logistic regression model was created for the binary dependent variable of whether a person was accepted into any MD-PhD program. The independent variables for the model were number of papers, number of presentations, family income quintile, race/ethnicity, parental education (no college, college, or graduate degree), parental medical education (no medical degree, MD or DO, or MD-PhD), and reapplicant status. The application year was included as a model covariate to adjust for differences in acceptance rates over time. All analyses were performed using the open-source statistical software language R (37, 38). ChatGPT was used to troubleshoot some code (39). A *P* value of less than 0.05 was considered significant.

## Author contributions

DKAW and BC are responsible for access to data and integrity of the work. DKAW and BC were responsible for concept and design of the study. DKAW, BC, TK, RK, MCG, AA, CS, and CO were responsible for acquisition, analysis, or interpretation of data. DKAW, BC, TK, RK, MCG, AA, CS, CO, JO, JPB, and MS drafted the manuscript. DKAW, BC, TK, RK, MCG, AA, CS, CO, JO, JPB, MS, MHA, and DD provided critical revision of the manuscript for important intellectual content. TK, RK, and MCG were responsible for data cleaning and visualization. TK, RK, MCG, BC, and DKAW performed statistical analysis. DKAW, BC, TK, RK, MCG, and MS provided administrative, technical, or material support. DKAW and BC obtained funding: Most of the design, data generation, data analysis, interpretation, and drafting of the manuscript was a collaboration between the two co–first authors, DKAW and BC. We have carefully considered the order of authorship, which was agreed upon and modified during planning and execution of the study. DKAW is designated the first co–first author because he had the original idea for this study.

## Figures and Tables

**Table 1:**
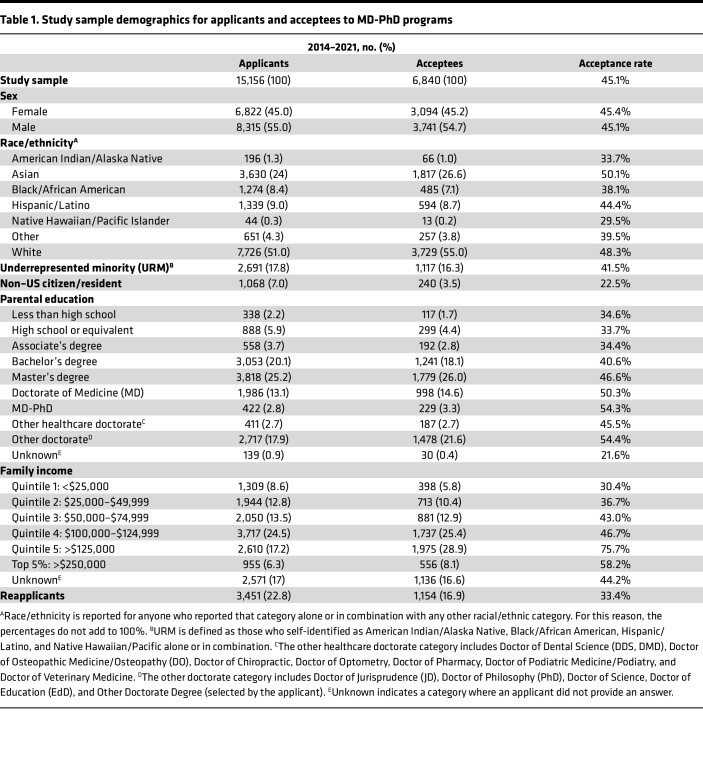
Study sample demographics for applicants and acceptees to MD-PhD programs

**Table 2:**
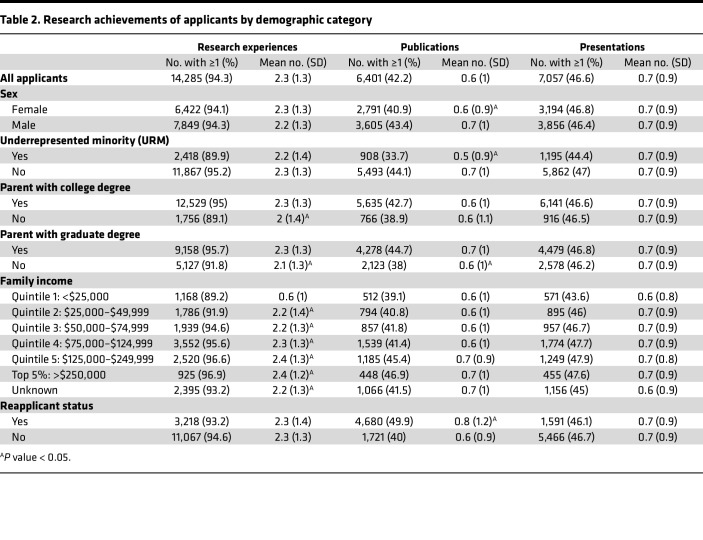
Research achievements of applicants by demographic category

**Table 3:**
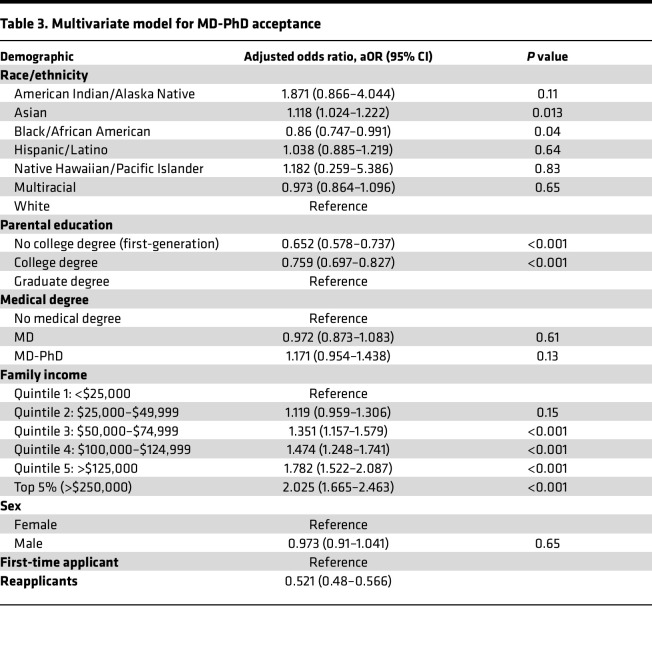
Multivariate model for MD-PhD acceptance

## Version 1 02/08/2024

Electronic publication
